# Linkage disequilibrium and inbreeding estimation in Spanish Churra sheep

**DOI:** 10.1186/1471-2156-13-43

**Published:** 2012-06-12

**Authors:** Elsa García-Gámez, Goutam Sahana, Beatriz Gutiérrez-Gil, Juan-Jose Arranz

**Affiliations:** 1 Dpto. Producción Animal, Universidad de León, 24071, León, Spain; 2 Department of Molecular Biology and Genetics, Aarhus University, Aarhus, Denmark

## Abstract

**Background:**

Genomic technologies, such as high-throughput genotyping based on SNP arrays, have great potential to decipher the genetic architecture of complex traits and provide background information concerning genome structure in domestic animals, including the extent of linkage disequilibrium (LD) and haplotype blocks. The objective of this study was to estimate LD, the population evolution (past effective population size) and the level of inbreeding in Spanish Churra sheep.

**Results:**

A total of 43,784 SNPs distributed in the ovine autosomal genome was analyzed in 1,681 Churra ewes. LD was assessed by measuring *r*^*2*^ between all pairs of loci. For SNPs up to 10 kb apart, the average *r*^*2*^ was 0.329; for SNPs separated by 200–500 kb the average *r*^*2*^ was 0.061. When SNPs are separated by more than 50 Mbp, the average *r*^*2*^ is the same as between non-syntenic SNP pairs (0.003). The effective population size has decreased through time, faster from 1,000 to 100 years ago and slower since the selection scheme started (15–25 generations ago). In the last generation, four years ago, the effective population size was estimated to be 128 animals. Inbreeding coefficients, although differed depending on the estimation approaches, were generally low and showed the same trend, which indicates that since 2003, inbreeding has been slightly increasing in the studied resource population.

**Conclusions:**

The extent of LD in Churra sheep persists over much more limited distances than reported in dairy cattle and seems to be similar to other ovine populations. Churra sheep show a wide genetic base, with a long-term viable effective population size that has been slightly decreasing since selection scheme began in 1986. The genomic dataset analyzed provided useful information for identifying low-level inbreeding in the sample, whereas based on the parameters reported here, a higher marker density than that analyzed here will be needed to successfully conduct accurate mapping of genes underlying production traits and genomic selection prediction in this sheep breed. Although the Ovine Assembly development is still in a draft stage and future refinements will provide a more accurate physical map that will improve LD estimations, this work is a first step towards the understanding of the genetic architecture in sheep.

## Background

The application of recently developed genomic technology, such as genome-wide SNP genotyping has great potential to increase our understanding of the genetic architecture of complex traits and to improve selection efficiency in domestic animals through genomic selection. However, the success of these approaches depends on the extent of the linkage disequilibrium (LD) across the genome, which may vary between populations. As an example, the extent of linkage disequilibrium serves to assess the number of markers required to associate genetic variation with economically important traits. A population with extensive LD will require a lower marker density; in contrast, if LD persists over short distances many more markers will be required to obtain the same power to detect association [1]; the same reasoning could be applied to genomic selection efficiency [2,3]. Similarly, the signatures of genomic regions under positive selection can be identified by studying the haplotype block structure throughout the genome [4].

The linkage disequilibrium pattern can also provide insight into the evolutionary history of a population. The extension of LD in the genome could be used to infer ancestral effective population size (*N*_*e*_) [5-7]. This is an important population parameter that helps to explain how populations evolved and can improve the understanding and modeling of the genetic architecture underlying complex traits [8].

Another aspect of interest while studying a commercial population under selection pressure is to study the level of inbreeding. Traditional estimation of the inbreeding coefficient based on pedigree data [9] is dependent on the completeness and accuracy of the available pedigree records. Currently, using the information provided by molecular markers (genome-wide SNP chip panels), we can estimate this coefficient with or without pedigree information [10]. Several methods have been described for this purpose [10-13].

An increasing number of studies have analyzed LD features in livestock species, especially in cattle [4,14,15] but also in pigs [16], horses [17] and chicken [18]. In domestic sheep, LD studies based on microsatellite data [1,19] found extended LD across the genome, although a marked variation between different breeds was reported [1]. Within the framework of the SheepHapMap project, the Illumina Ovine SNP50 BeadChip has been used to present a preliminary evaluation of LD in 74 diverse breeds [20]. A subset of informative SNPs from this chip has also been utilized in wild sheep to calculate the extent of LD and evaluate the usefulness of this chip, which was developed for domestic sheep, for conducting genome-wide association studies in wild sheep populations [21].

The objective of this study was to characterize LD in a Spanish Churra sheep commercial population using data generated with the Illumina Ovine SNP50 BeadChip. This genomic tool is currently being used in this dairy sheep breed to fine-map previously reported QTLs [22] and to obtain a preliminary assessment of the genomic selection approach [23]. Thus, we have studied the genome structure (LD and haplotype blocks), population evolution (past effective population size) and the level of inbreeding present in this population, which will provide fundamental information on the genome organization of this Spanish sheep breed.

## Methods

### Resource population and SNP genotyping

A commercial population of Spanish Churra sheep was analyzed in this research. Blood samples were collected from 1,710 Spanish Churra ewes belonging to 16 half-sib families and distributed across 20 different flocks. Semen straws were obtained for the 16 sires. The use of animals was performed in compliance with the guidelines approved by the University of Leon ethical commission.

DNA was extracted from blood and semen samples using standard protocols, as described in [24]. A control for Mendelian inheritance errors was performed at this stage using a panel of 18 microsatellite markers [25]. Finally, a total of 1,696 DNA samples with a concentration of 50 ng/μl and A_260/280_ ratio above 1.8 were used for Illumina Ovine SNP50 BeadChip genotyping. Genotyping was performed commercially at AROS Applied Biotechnology AS (Aarhus, Denmark) and LABOGENA (Jouy-en-Josas, France). Quality control (QC) of the raw genotypes consisted of checking the GenCall Score (GCscore) using the GenomeStudio software (Illumina Inc. *San DiegoCA*). Genotypes with a GCscore < 0.15 were set as missing genotypes.

### Quality control, marker order and genetic distances

The SNPs included in the Illumina Ovine SNP50 BeadChip were mapped using the Ovine Genome Assembly v2.0 [26]. The markers were filtered to exclude loci assigned to unmapped contigs. Only the SNPs located on the sheep autosomes were considered in further analyses.

We performed QC of the genotypes in two stages, first implementing the control on a ‘per-individual’ basis prior to conducting QC on a ‘per-marker’ basis to maximize the number of markers that remained in the study [27,28]. First, individuals were removed if they had more than 10% missing genotypes. Secondly, the marker-QC included three steps: (i) control of call rate (≥ 0.95), (ii) minor allele frequency (MAF) (≥ 0.05) and (iii) correspondence with Hardy-Weinberg equilibrium (HWE) (p-value > 0.00001). For the markers that passed the previously mentioned QC, we performed a final QC using the available pedigree. Thus, the genotypes causing Mendelian inheritance inconsistencies were set to “missing” and afterwards inferred based on the sire’s genotype and the population frequencies of the two possible alleles. This imputation process was done with an unpublished FORTRAN based program (VerifTyp 1.0; Boichard D and Druet T, personal communication), which performs 10 inference iterations where the base population frequencies are re-estimated at each step depending on the reconstruction of genotypes. A probability threshold was set to avoid over-representation of very frequent alleles.

The initial locus order between adjacent markers, which was based on the Sheep Genome Assembly v2.0, was assessed using the *fixed* option of a modified version of CRI-MAP [29], v2.503 (kindly provided by J. F. Maddox). The information derived from this control was used to mend some colocation problems, as some markers had more than one hit in the reference assembly (B. Dalrymple, personal communication). The resulting marker order and positions were used as the physical map to perform the LD analyses Additional file 1.

### Haplotype construction

The ideal scenario to measure the extent of LD within a population is to analyze “non-related” individuals. Our resource population of half-sib families had initially been selected to perform linkage-based QTL mapping studies using a daughter design [30], and therefore, the sampled individuals were related. To overcome this limitation, we attempted to obtain a representation of independent haplotypes of the population under study. With this purpose, we calculated chromosome phases taking into account the population pedigree structure using PHASEBOOK package [31]. Following the three-step approach described by the authors, we first used the LinkPHASE 2.3 program (part of PHASEBOOK) to obtain partially phased genotypes using pedigree and linkage information (steps I and II). Then, DAGPHASE 1.1 (part of PHASEBOOK), in combination with BEAGLE 3.3 [32], was used to impute missing markers based on linkage disequilibrium (step III). For this analysis, we used i) DAGPHASE 1.1 option 1 to fill-in the missing base haplotypes at random, ii) 15 iterations using BEAGLE 3.3 to construct the optimal directed acyclic graph (DAG) and DAGPHASE 1.1 option 2 to sample the missing alleles of the base haplotypes according to a Hidden Markov Model (HMM) and iii) DAGPHASE 1.1 option 3 and the last DAG to calculate the haplotypes.

### Linkage disequilibrium and effective population size

Reconstructed haplotypes were selected to not have an overrepresentation of the sires’ haplotypes [15]. Sire haplotypes and maternal-inherited dam haplotypes were inserted into HAPLOVIEW v4.1 [33] to estimate LD statistics based on pairwise SNPs. For easy comparison of results with other reports, the two most commonly used statistics, *D’*[34] and *r*^*2*^[35], were computed for this study. For non-syntenic SNPs, a subset was used to estimate LD across the genome. This selection was based on a random representative sample of the SNPs analyzed in each chromosome (5% of the SNPs used in the analysis). Both LD metrics (*D’* and *r*^*2*^) were estimated for each non-syntenic pair. To assess how far LD extends, we average *r*^*2*^ based on the SNP distance in 1-Mb intervals and calculated the half-length of *r*^*2*^[21]. This half-length is the distance at which LD decays to half of its maximum value [36].

HAPLOVIEW v4.1 was also used to define the haplotype blocks present in the genome. The method followed for block definition was previously described by Gabriel et al. [37]. A pair of SNPs is defined to be in ‘strong LD’ if the one-sided upper 95% confidence bound of *D’* is higher than 0.98 and if the lower bound is above 0.7. In contrast, ‘strong evidence for historical recombination’ is defined if the upper confidence bound on D’ is less than 0.9 [37].

Past effective population size (*N*_*e*_) was calculated for 11 time points. Based on the physical map used for the LD analysis, genetic distances between adjacent markers were calculated using three conversion rates: (i) considering a 1 cM ~ 1 Mb conversion rate for all the chromosomes, (ii) considering the specific cM/Mb ratio calculated for each chromosome by comparing the genetic and physical map in the CRI-MAP analysis and (iii) considering the average conversion rate estimated across the genome for all the chromosomes. Each genetic distance (*c,* in Morgans) corresponds to a value of *T* generations in the past. This value was calculated as *T = 1/(2c)*. The following formula was used to estimate *N*_*e*_[8]:

(1) $$r^{2}=1/\left(1+4N_{e}c\right)+1/n\text{,}$$

where *c* is the distance in Morgans, and *n* is the chromosome sample size (number of haplotypes) used in the analysis. According to the genetic distances between markers, SNP pairs were stacked into bins of 1,000 pairs, and the average distance and *r*^*2*^ estimated for each bin were used to calculate the *N*_*e*_.

### Inbreeding coefficients

Two different approaches were used to estimate the coefficient of inbreeding (*F*) within the Spanish Churra population. The pedigree-based *F* (*F*_*PED*_) was estimated using the Relax2 software [38], based on the algorithm described by Meuwissen and Luo [39] using available pedigree records since 1978 (5,956 animals in total). Marker-based inbreeding coefficients were estimated using the GCTA software [12]. To calculate the marker-based *F* values, we used the population under study as the base population. Allele frequencies were estimated across the 1,681 animals, and the GCTA software was used to obtain *F* values. Three different metrics were obtained using the *--ibc* option of the program: a) based on the variance of the additive genotype (*F*_*1*_), b) based on the excess of homozygosity (*F*_*2*_) and c) based upon the correlation between uniting gametes (*F*_*3*_) [12].

## Results

### SNP distribution and frequencies

Out of a total of 54,241 SNPs genotyped in this study, 1,516 SNPs were unmapped, and 215 were located on sex chromosomes as per Ovine Genome Assembly v2.0. Thus, 52,510 SNPs mapped onto the 26 sheep autosomes were used in the described analyses.

**Figure 1:**
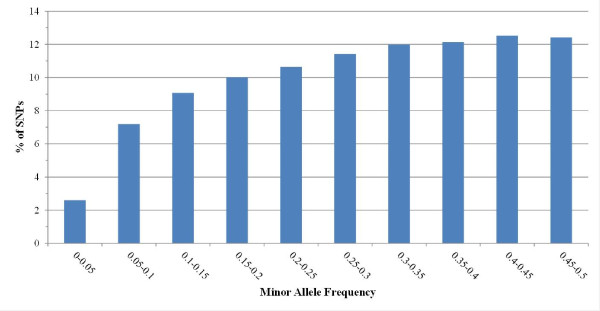
**The distribution of SNPs across the genome as a function of the minor allele frequency (MAF).** The percentage of SNPs that passed quality control (QC) for different MAF bins is depicted here.

Of the 1,696 genotyped animals, 15 individuals did not pass the QC. Thus, a total of 1,681 animals were used in the analyses. The number of markers removed during QC was 8,726 SNPs: 4,140 SNPs were deleted due to low call rate (< 0.95); 3,044 SNPs did not reach minimum MAF (< 0.05); and 1,542 markers were not in HWE (P ≤ 0.00001). The total number of markers used in the analyses was 43,784 SNPs. The distribution of these SNPs per chromosome is described in Table 1, ranging from 598 on OAR24 to 4,987 on OAR1. The average distance between SNPs was 55.74 kb, ranging from 51.47 kb in OAR9 to 69.71 kb in OAR24. The distribution of MAF across the chromosomes was similar, with a mean value of 0.288 (Table 1). Figure 1 represents the distribution of SNPs in MAF bins. Around 50% of the SNPs had an MAF value over 0.3. The cM/Mb ratios calculated for each chromosome by comparing the genetic and physical maps in the CRI-MAP analysis ranged between 1.5 on chromosome 2 and 2.37 on chromosome 20 (Table 1). The average conversion rate estimated across the genome was 1.85 cM ~ 1 Mb.

**Table 1:** A summary of statistics for the SNPs that passed quality control (QC)

**Chromosome**	**NumSNP**	**Average Distance between SNPs (kb)**	**Average MAF**	**Average Heterozygosity**	**Rate cM/Mb**	**Average D’**	**Average r**^**2**^	**Average r**^**2**^** (<100Kb)**
1	4,987	55.38	0.287	0.352	1.55	0.127	0.006	0.151
2	4,676	53.45	0.284	0.345	1.5	0.138	0.008	0.171
3	4,164	53.78	0.288	0.351	1.64	0.132	0.008	0.164
4	2,246	52.76	0.287	0.352	1.75	0.151	0.010	0.163
5	1,978	54.26	0.290	0.349	1.71	0.161	0.012	0.157
6	2,190	53.22	0.291	0.354	2.04	0.166	0.013	0.166
7	1,887	52.84	0.293	0.354	1.72	0.144	0.009	0.139
8	1,717	52.91	0.282	0.346	1.62	0.151	0.011	0.170
9	1,836	51.47	0.283	0.346	1.71	0.148	0.009	0.150
10	1,523	54.86	0.294	0.356	1.61	0.155	0.012	0.180
11	963	64.12	0.292	0.355	2.12	0.154	0.012	0.151
12	1,456	54.36	0.296	0.358	1.64	0.148	0.011	0.145
13	1,402	59.14	0.283	0.347	1.85	0.150	0.009	0.155
14	959	64.89	0.277	0.344	2.15	0.162	0.010	0.142
15	1,374	58.59	0.291	0.356	1.54	0.139	0.010	0.163
16	1,312	54.33	0.282	0.346	1.86	0.158	0.011	0.144
17	1,178	61.54	0.291	0.354	1.88	0.158	0.011	0.160
18	1,192	56.35	0.292	0.358	1.83	0.152	0.010	0.146
19	1,032	58.76	0.279	0.344	2.05	0.158	0.009	0.147
20	910	55.46	0.301	0.361	2.37	0.170	0.015	0.145
21	724	66.68	0.277	0.339	1.94	0.164	0.010	0.138
22	942	53.60	0.284	0.353	2.06	0.158	0.010	0.126
23	934	66.93	0.296	0.358	1.82	0.167	0.013	0.149
24	598	69.71	0.303	0.366	2.22	0.152	0.013	0.153
25	846	52.05	0.295	0.355	2.02	0.173	0.014	0.134
26	758	57.91	0.281	0.347	2.05	0.169	0.014	0.148

### Linkage disequilibrium and haplotype blocks

**Figure 2:**
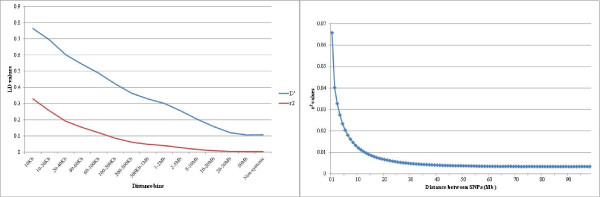
**Linkage disequilibrium (LD) across the genome as a function of genomic distance between markers is represented here for Spanish Churra sheep.A**) Average LD, measured using two parameters, *D’* and *r*^*2*^. SNP pairs were stacked according to their physical distance into 14 categories: < 10 kb, 10–20 kb, 20–40 kb, 40–60 kb, 60–100 kb, 200–500 kb, 0.5-1 Mb, 1–2 Mb, 2–5 Mb, 5–10 Mb, 10–20 Mb, 20–50 Mb or > 50 Mb; each point in the graph corresponds to one of these bins. **B**) Average LD (*r*^*2*^) binning SNPs into 1 Mb-interval categories; half-length LD (*r*^*2*^ = 0.033) approximate distance 2.5 Mb.

For the LD analysis, the total number of reconstructed ‘non-related’ haplotypes (chromosomes) was 1,692. A total of 42,381,374 syntenic pairs of SNPs was analyzed for all the autosomes. Average *D’* and *r*^*2*^ values, pooled over autosomes in different categories of map distances, are presented in Table 2. The distribution of both *D’* and *r*^*2*^ with respect to the physical distance separating loci is presented in Figure 2. As shown in Figure 2a and Table 2, there is a decline in *r*^*2*^ with increasing physical distance between SNPs: for SNPs up to 10 kb apart, the average *r*^*2*^ is 0.329; for SNPs separated by 200–500 kb, the average *r*^*2*^ is 0.061. When SNPs are separated by more than 50 Mbp, the average *r*^*2*^ (0.003) is the same as that found between non-syntenic SNP pairs (0.003). The distribution of *D'* is similar to that observed for *r*^*2*^. The half-length of *r*^*2*^ was 0.033, which corresponds to a distance between SNPs of 2.5 Mbp (Figure 2b). Small differences in average LD values were observed among chromosomes and they corresponded with differences in chromosome length (Table 1). Average *r*^*2*^ per chromosome ranged from 0.006 on OAR1 to 0.015 for OAR20. These differences across the genome were lower when comparing values obtained at the different distance bins. For example, average *r*^*2*^ values for SNPs up to 100 kb apart ranged from 0.126 on OAR22 to 0.180 on OAR10, which was the chromosome showing the highest level of LD between markers.

**Table 2:** Mean linkage disequilibrium among syntenic and nonsyntenic SNPs over different map distances

		**D’**				**r**^**2**^			
**Distance**	**NumPairs**	**Average**	**SD**	**Min**	**Max**	**Average**	**SD**	**Min**	**Max**
< 10 Kb	1,814	0.763	0.312	0.001	1	0.329	0.325	0	1
10-20 Kb	4,218	0.694	0.322	0.001	1	0.256	0.282	0	1
20-40 Kb	13,963	0.601	0.329	0	1	0.191	0.240	0	1
40-60 Kb	16,364	0.543	0.323	0	1	0.152	0.207	0	1
60-100 Kb	32,426	0.487	0.312	0	1	0.120	0.175	0	1
100-200 Kb	80,273	0.422	0.288	0	1	0.086	0.133	0	1
200-500 Kb	239,021	0.364	0.261	0	1	0.061	0.098	0	1
500 Kb-1 Mb	395,047	0.328	0.243	0	1	0.049	0.078	0	0.987
1-2 Mb	781,115	0.301	0.228	0	1	0.040	0.066	0	0.898
2-5 Mb	2,288,329	0.255	0.203	0	1	0.028	0.048	0	0.953
5-10 Mb	3,644,862	0.203	0.170	0	1	0.017	0.030	0	0.747
10-20 Mb	6,661,473	0.158	0.140	0	1	0.009	0.016	0	0.661
20-50 Mb	15,118,487	0.120	0.114	0	1	0.005	0.008	0	0.530
> 50 Mb	13,103,982	0.106	0.106	0	1	0.003	0.006	0	0.229
Non-syntenic	2,257,088	0.107	0.106	0	1	0.004	0.006	0	0.169

Average values for *D’* and *r*^*2*^ pooled over autosomes in different categories; minimum, maximum and standard deviation are shown in this table. The same parameters for non-syntenic SNP pairs are also displayed.

A summary of the distribution, size, number and SNPs involved in the haploblocks per chromosome are presented in Table 3. A total of 2,099 haploblocks spanning 56,726 kb (2.32%) of the Churra autosomal genome were detected. The average block size was 27.03 kb, ranging from 0.04 kb (OAR14, 2 SNPs) to 1,263 kb (OAR2, 8 SNPs). In total, 4,780 SNPs (10.92% of all SNPs used) formed blocks with a range of 2–13 SNPs per tract. The chromosomes showing the longest and shortest haplotypic structures in the genome were OAR2 with 252 blocks spanning 11,149 kb and OAR26 with 22 blocks covering 367 kb. There was a region in OAR10, from 34.2 to 42.0 Mbp, with a high density of haplotype blocks. This region included 8 haploblocks involving the 61% of haplotype block length of this chromosome (3,197 kb of the total of 83,632 Kb). The length of these blocks varied between 2 SNPs, for one of the haploblocks and 9 SNPs in three of the other haploblocks.

**Table 3:** Block structure per chromosome

**Chromosome**	**Number of blocks**	**Total block length (Kb)**	**% of Chromosome length in blocks**	**Min. block length (Kb)**	**Max. block length (Kb)**	**Number of SNPs in blocks**	**% of SNPs in blocks**
1	257	5,440.86	1.97	1.74	265.59	569	11.41
2	252	11,148.79	4.46	3.92	1,262.79	634	13.56
3	200	5,321.47	2.38	2.79	385.36	464	11.14
4	103	3,273.46	2.76	2.89	228.94	244	10.86
5	86	2,023.86	1.88	0.07	325.55	191	9.66
6	115	2,705.20	2.31	3.72	225.92	257	11.74
7	95	1,753.56	1.75	4.06	158.77	204	10.81
8	88	2,256.32	2.49	2.62	262.30	198	11.53
9	109	2,886.37	3.04	3.66	472.64	249	13.56
10	79	5,231.93	6.26	2.46	1,075.88	210	13.79
11	41	779.45	1.25	5.76	177.74	88	9.14
12	80	1,154.64	1.46	1.95	182.06	165	11.33
13	71	1,725.72	2.08	3.75	175.94	161	11.48
14	34	648.62	1.04	0.04	120.50	76	7.92
15	62	1,716.25	2.12	2.13	248.80	140	10.19
16	52	980.30	1.37	2.46	134.49	113	8.61
17	42	1,225.16	1.69	2.99	231.71	103	8.74
18	50	854.06	1.25	5.08	152.75	107	8.98
19	51	978.44	1.61	5.73	316.69	105	10.17
20	33	660.86	1.31	6.33	155.29	72	7.91
21	34	521.40	1.08	2.84	116.56	73	10.08
22	48	870.01	1.72	2.63	200.82	104	11.04
23	39	1,009.87	1.61	6.71	328.76	85	9.10
24	19	453.21	1.09	4.38	233.82	40	6.69
25	37	738.85	1.68	6.76	205.07	82	9.69
26	22	366.91	0.83	0.92	128.69	46	6.07
All	2,099	56,725.56	2.32	0.04	1,262.79	4,780	10.92

The number of blocks, total, minimum and maximum block length, percentage of the sequence covered by blocks, number and percentage of SNPs in blocks on a per chromosome basis are reported in this table. Values obtained across the genome are also shown.

### Past effective population size estimations

**Figure 3:**
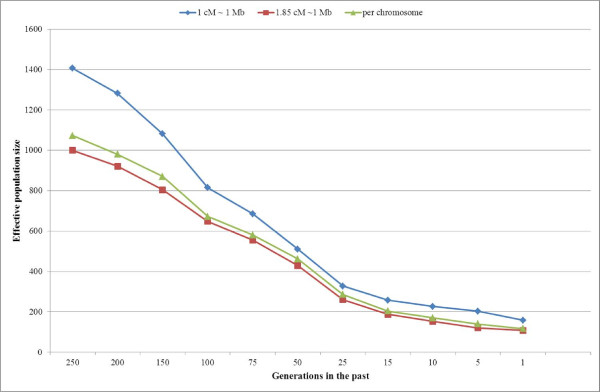
**Past effective population size (*N***_***e***_**) over the past generations based on linkage disequilibrium calculations from 26 autosomes.** We estimated *N*_*e*_ from the average *r*^*2*^ at different marker distances using three different cM/Mb conversion rates: a) 1 cM ~ 1 Mb (red), b) 1.85 cM ~ 1 Mb (blue) or c) individual rates for each chromosome, as indicated in Table 1 (green). Data points were based on at least 1,000 marker pairs.

A graphical representation of the *N*_*e*_ values at each time point, from 250 to 1 generation ago, and for each of the three different cM/Mb conversion rates used to calculate genetic distance between markers is given in Figure 3. Taking into account a generation interval of 4 years, these results correspond to Churra populations 1,000 to 4 years ago, approximately. The results show that *N*_*e*_ has decreased through time, faster at 1,000 to 100 years ago and slower since selection scheme started (15–25 generations ago) (Figure 3). The effective population size in the last generation (4 years ago) averaged across three cM/Mb ratios is calculated to be 128 animals.

### Inbreeding measurement

**Figure 4:**
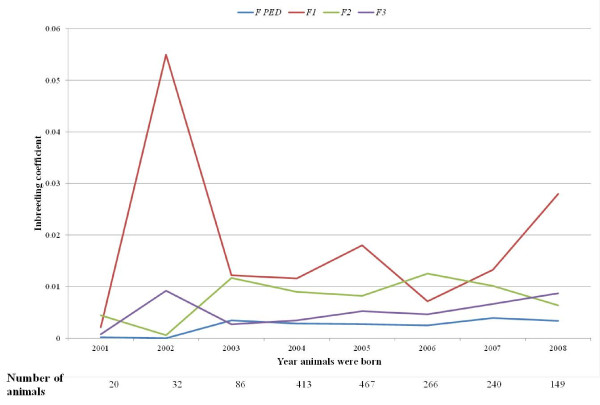
**Average inbreeding coefficients for animals born from 2001 to 2008. Average values of the positive inbreeding coefficients calculated using pedigree (*F***_***PED***_**) or marker information (*F***_***1***_**,*F***_***2***_**,*F***_***3***_**) are represented in this figure.** The three marker-based metrics represented here are based on the variance of the additive genotype (*F*_*1*_), the excess of homozygosity (*F*_*2*_) and the correlation between uniting gametes (*F*_*3*_). The number of animals used in each time point is listed below the X-axis.

Available pedigree used in this analysis included 5,956 animals, which represents a pedigree depth of 6 generations. From the 11,912 expected parents, 41% were missing data. The inbreeding coefficient calculated using this pedigree information, *F*_*PED*_, was estimated to be null (no inbreeding) for 5,856 out of the 5,956 animals, with an average of 0.001. For the remaining 100 ‘inbred animals’, the mean value was 0.064. To compare these results with marker-based inbreeding values, we extracted pedigree-based inbreeding coefficients for the 1,681 genotyped animals. For these animals, a mean value of 0.003 was obtained. Using molecular information, we did not obtain any ‘non-inbred’ animal (*F* = 0), but some results were negative. With the aim of comparing results between the marker-based and the pedigree-based methods, we transformed negative values to 0 and studied the differences in values across time. Average values for the positive estimates were 0.015 (*F*_*1*_), 0.009 (*F*_*2*_) and 0.005 (*F*_*3*_). Figure 4 shows the inbreeding coefficients obtained for animals born each year since 2001. A peak for the values obtained using the *F*_*1*_ method was found for year 2002. A more detailed study of the animals born that year showed more than half of these animals had an inbreeding coefficient higher than 0.0625, which is very high according to the results obtained across the population. Although the estimates from various approaches were different, they all show the same trend and indicate that in the studied resource population inbreeding has increased slightly since 2003.

## Discussion

This study presents an analysis of the extent of LD in Spanish Churra sheep using 43,784 SNPs distributed across the autosomal genome, although the draft stage of the version of the Ovine Assembly it is based on should be taken into account. Future refinements and updates in the physical maps can lead to changes in the estimations reported here. To enable comparison with previous studies in sheep and other domestic species, we estimated two pairwise statistics: *D’* and *r*^*2*^. *D’* values were higher than those estimated for *r*^*2*^. This might be because rare alleles and unobserved haplotypes tend to inflate *D’* but not *r*^*2*^[1].

Comparing the level of LD obtained in different studies is difficult because of different sample sizes, LD measures, marker types, marker densities and recent and historical population demographics [4]. Previous reports in sheep based on microsatellite marker analysis have described LD as extensive (up to 20 cM) [1,19], although its magnitude and significance was shown to vary markedly between different breeds [1]. The results reported for wild sheep [21] also showed LD extended over long distances (half-length *r*^*2*^ of 4.6 Mb), which contrasts with the short extension of LD reported here for Churra sheep (half-length *r*^*2*^ value 2.5 Mb). A recent assessment of LD based on the analysis of 51,446 SNPs in a sample of Sarda rams showed a similar level of LD than in Churra sheep, with an average *r*^*2*^ value over 1,000 kb of 0.072 [40]. Compared with the results based on SNP genotyping described for cattle [4,14,15,41], LD estimates between syntenic and nonsyntenic loci in Churra sheep was two times lower. Initial results from the analysis of 74 domestic sheep breeds with the Illumina Ovine SNP50 BeadChip [20] were in concordance with our findings, which suggests a relatively low level of LD in sheep and a substantially lower LD in sheep when compared with a wide range of cattle breeds, including dairy and beef cattle [42]. This analysis also showed Churra sheep as one of the breeds with a more remarkable decay of LD with the distance between markers when compared with other breeds [20]. Average *r*^*2*^ between nonsyntenic SNP pairs provides an idea of the LD that can be expected by chance. None of the nonsyntenic SNP pairs tested showed a ‘high’ LD value (*r*^*2*^ > 0.25).

Differences in LD between chromosomes have already been reported in Holstein cattle [4,15]. These can be attributed to recombination rates varying between and within chromosomes, heterozygosity, genetic drift and effects of selection [4]. Our results for average LD within a chromosome are in concordance with the block structure across the genome. Chromosomes showing higher LD also have more and longer blocks than chromosomes with lower average LD. In Churra, 88% of the blocks contained just two SNPs. Preliminary results from the SheepHapMap project also identified an overall limited genome coverage in haplotype blocks (of at least three SNPs) for domestic breeds with Churra showing the lowest coverage (0.8%), whereas wild Soay sheep showed a large genome coverage (21.84%) [20]. Compared with domestic sheep, the genomic distribution and coverage of the genome by haplotype blocks is higher in other species, such as cattle [4,43], as expected according to the higher LD between markers reported in these species.

Also within the framework of the SheepHapMap project genomic regions containing signals of selection have been identified across a wide range of sheep breeds [44]. Higher homozygosity and LD is expected in regions that have undergone selection and are now fixed in the breed under study. Also, more and longer haplotype blocks are expected in those regions. Although there were haplotype blocks close to some of the regions related to selection, none of the high-Fst SNPs depicted by those authors [44] were involved in blocks in Spanish Churra sheep. For example, the region of OAR10 containing 8 haploblocks in Churra sheep is close to the *polled* locus (*RXFP2* gene), which is related to the presence of horns in sheep [44]. However, none of the SNPs linked to the polled phenotype were included in the Churra haploblocks. The longest haplotype block across the genome found in this study, which involved 8 SNPs and was located in OAR2 (111.9 – 113.1 Mbp), comprises the *HERC2* gene, which has already been related to pigmentation in cattle [45].

We also investigated the past effective population size (*N*_*e*_) in the Churra sheep commercial population under study. First historical references from the existence of Spanish Churra sheep date from Middle Ages (thirteenth century) approximately 800 years ago [46]. Therefore, the time points chosen in this work were based on this historical information. The correlation between the results of the three different cM/Mb calculations was over 0.99. Major differences between the estimates based on the three different ratios are found at small distances, corresponding to more than 75 generations ago (Figure 3). Changes in the effective population size reflect past events that occurred in the corresponding populations. In Spanish Churra, the *N*_*e*_ value has been descending through time until the selection scheme began. From that point on, no major changes are found. Effective population size estimated 50 generations ago in Churra (*Ne* = 467 animals) is in agreement with the observations reported within the framework of the SheepHapMap project, where most of the sheep breeds displayed high *N*_*e*_, and only two populations showed a narrow genetic base comparable to the current *N*_*e*_ of domestic cattle breeds (*N*_*e*_ < 150) [44]. No other *Ne* estimations have been reported so far in sheep. High selection pressure and the use of artificial insemination are the main reasons for the low Ne values obtained in cattle [42]. To ensure an animal population is long-term viable, a threshold of *Ne* = 100 has been given [47]. Our results of current effective population size (*Ne* = 128) are above the threshold, but care should be taken on this regard to ensure that the effective population size is maintained.

The LD estimates reported in this work can serve to assess the utility of the Ovine SNP50 Beadchip to address fine-mapping studies in Churra sheep. In cattle, McKay et al. [48] showed that at a physical distance of 100 kb separating flanking SNP loci, the average *r*^*2*^ was 0.15-0.2; considering a bovine genome length of 2.87 Gb, they concluded that 28,700 fully informative markers would be needed to saturate the cattle genome at an average resolution of 100 kb. Considering the lower value of LD estimates reported in this study, one can easily estimate that to obtain similar resolution in Churra sheep the marker density needs to be at least two times higher than the currently analyzed dataset. Hence, to implement genomic selection in this population with appropriate accuracy, a SNP array of higher density would be valuable. In this regard, following previous reported estimations [2,3] we can estimate how many SNPs will be needed to accurately estimate breeding values in Churra sheep. Considering a marker density of 20 SNPs per genome effective segment, which represents each independent chromosome segment [3], a population with *Ne* of 128 animals and a genome of 30 Morgans, the SNP chip should include approximately 95,000 SNPs (assuming the same percentage of successful genotyping obtained in this study) to improve the accuracy of genetic breeding values estimation.

Pedigree-based inbreeding calculations rely on the completeness and accuracy of the available pedigree. The results reported herein based on the available Churra pedigree showed 94% of the animals included in the analysis were ‘non-inbred’, although this is due to the lack of a deep pedigree. We obtained some negative values for inbreeding coefficients, which corresponded to animals with lower homozygosity than the average population. This could be because we estimated the allele frequencies from the currently genotyped population instead of the base population. Correlation between the different methods to estimate inbreeding ranged from 0.27 (*F*_*1*_*vs. F*_*2*_) to 0.83 (*F*_*2*_*vs. F*_*3*_), with *F*_*1*_ as the most different. In general, values calculated using pedigree information were lower than those obtained through marker analysis. The latter could be inflated because we assumed a homogeneous population [13], while there is a structure due to the experimental design of the linkage-based mapping studies for which the resource population had initially been selected. Comparing between the three marker-based methods, a different percentage of the analyzed animals showed an inbreeding value higher than the critical level (6.25%, obtained when mating cousins, [49]). This proportion varied from 8.45% (*F*_*1*_) to 1.6% (*F*_*3*_), which is lower than that described for Finnsheep [49]. This percentage was very high when analyzing results from 2002 (method *F*_*1*_), 50% of the animals had an *F*_*1*_ > 0.0625. Moreover, we were not able to compare between methods as most of the animals for *F*_*2*_ and *F*_*3*_ had negative values which might also explain the low correlation found between *F*_*1*_ and *F*_*2*_. In agreement with previous studies, the results presented here show that genomic data sets can provide useful information on a per sample basis in cases of complex genealogies or in the absence of genealogical data [13].

## Conclusions

In the studied Churra sheep population, LD decayed with increasing genomic distance and the analysis yielded similar values than previously reported in other sheep populations. An estimation of genetic distance from physical position showed few differences between chromosomes, which did not affect the past effective population size results. Effective population size seems to have been decreasing until the recent selection scheme in Spanish Churra began. Marker information aided the estimation of the level of inbreeding present in the sample, although more accurate results could be obtained if we had a base population to estimate allele frequencies. In conclusion, the results reported herein are a first step toward understanding the genomic architecture of a domestic sheep breed and can be used to access the feasibility of direct future selection based on genomic data. The level of LD estimated in Churra sheep indicates that the present Illumina Ovine SNP50 BeadChip is not an optimum and the future availability of a high-density SNP-array or the use of next-generation sequencing methods will improve the performance of QTL fine-mapping studies and genomic selection accuracy in this population.

## Competing interests

The authors declare that they have no competing interests.

## Author’s contributions

EG-G performed the quality control, analyses and drafted the manuscript. BG-G and GS participated in the design and coordination of the study and helped to draft the manuscript. JJA conceived of the study and participated in its design and coordination. All authors have read and approved the final manuscript.

## Supplementary Material

Additional file 1 **Physical and linkage map information used in the analysis.** This file contains information about physical and genetic position of the markers included in this analysis. This table contains three columns: SNP identification, chromosome, physical position (in base pairs) and genetic position (in cM) obtained using the *fixed* option of a modified version of CRI-MAP v2.503 (kindly provided by JF Maddox). Click here for file
